# Patients with Congenital Systemic-to-Pulmonary Shunts and Increased Pulmonary Vascular Resistance: What Predicts Postoperative Survival?

**DOI:** 10.1371/journal.pone.0083976

**Published:** 2014-01-08

**Authors:** Hui-Li Gan, Jian-Qun Zhang, Qi-Wen Zhou, Lei Feng, Fei Chen, Yi Yang

**Affiliations:** Department of Cardiac Surgery, Beijing Anzhen Hospital, Capital Medical University, Beijing Institute of Heart, Lung and Blood Vessel Diseases, Beijing, China; Tabriz University of Medical Sciences, Iran (Islamic Republic of)

## Abstract

**Background:**

We carried out a retrospective data review of patients with systemic to pulmonary shunts that underwent surgical repair between February 1990 and February 2012 in order to assess preoperative pulmonary vascular dynamic risk factors for predicting early and late deaths due presumably to pulmonary vascular disease.

**Methods and Results:**

A total of 1024 cases of congenital systemic-to-pulmonary shunt and advanced pulmonary vascular disease beyond infancy and early childhood were closed surgically. The mean follow up duration was 8.5±5.5 (range 0.7 to 20) years. Sixty-one in-hospital deaths (5.96%, 61/1024) occurred after the shunt closure procedure and there were 46 late deaths, yielding 107 total deaths. We analyzed preoperative pulmonary vascular resistance index (PVRI), pulmonary vascular resistance index on pure oxygen challenge (PVRIO), difference between PVRI and PVRIO (PVRID), Qp∶Qs, and Rp∶Rs as individual risk predictors. The results showed that these individual factors all predicted in-hospital death and total death with PVRIO showing better performance than other risk factors. A multivariable Cox regression model was built,and suggested that PVRID and Qp∶Qs were informative factors for predicting survival time from late death and closure of congenital septal defects was safe with a PVRIO<10.3 WU.m^2^ and PVRID>7.3 WU.m^2^ on 100% oxygen.

**Conclusions:**

All 4 variables, PVRI, PVRIO, PVRID and Qp∶Qs, should be considered in deciding surgical closure of congenital septal defects and a PVRIO<10.3 WU.m^2^ and PVRID>7.3 WU.m^2^ on 100% oxygen are associated with a favorable risk benefit profile for the procedure.

## Introduction

Cardiac defects are among the most common causes of congenital disease with atrial and ventricular intracardiac shunts accounting for a significant proportion of such malformations. Preoperative pulmonary vascular disease is an important risk factor for death or right-heart failure in older patients undergoing palliative surgical repair for intracardiac shunting lesions. Despite many published reports, it remains unclear which preoperative hemodynamic variables best predict a satisfactory surgical outcome, i.e., acceptably low pulmonary vascular resistance (PVR) after operation [1], [2]. Previous papers report a relatively small number of patients, a serious limitation given the substantial variation in the pulmonary vascular response to increased pressure and flow. Postoperative follow-up is limited in most previous reports, which becomes a significant issue over time as PVR may increase years after operation. Moreover, few reports have been published that present the results of studies designed to determine the risk factors (using multivariate analysis) affecting the outcome of the surgical procedures to treat intracardiac shunts. This has led to a lack of clear guidelines for all surgical centers, especially those in parts of the world where surgeons have to deal with a large population of untreated older patients with congenital heart disease (CHD) and elevated PVR.

Surgical interventions for CHD have allowed long-term survival despite incomplete elimination of shunting. However, whether pulmonary vascular hemodynamic parameters could predict in-hospital death or late death in surgical patients with intracardiac shunts remain ill defined. Here, we carried out a retrospective data review of patients with systemic to pulmonary shunts that underwent surgical repair over a 10-year span between February 1990 and February 2012 in order to assess preoperative pulmonary vascular dynamic risk factors for predicting early and late deaths due presumably to pulmonary vascular disease.

## Methods

The Ethics Committee of Beijing Anzhen Hospital approved this retrospective study and written informed consent was obtained from each patient or his or her legal surrogate for the operation. Because of the retrospective nature of this study, no patient consent was required; it was specifically waived by the approving IRB.

### Patients

We retrospectively reviewed the demographic, clinical and surgical data of patients who underwent surgical repair for congenital intracardiac shunts at Beijing Anzhen Hospital over a 10-year span between February 1990 and February 2012. A patient was excluded from the analysis if 1) he or she also received heart valve repair or replacement, or other cardiac surgical procedures; 2) he or she had a residual heart defect after surgery, which may have impacted the severity of residual pulmonary hypertension; 3) he or she had defects such as branch pulmonary arterial stenosis, or obstruction of isolated pulmonary veins that preclude accurate calculation of PVR, Qp∶Qs, and Rp∶Rs. To determine surgical operability, all patients were discussed at a multidisciplinary team meeting consisting of pulmonary hypertension specialists, radiologists and cardiac surgeons. Closure of the defect was carried out for patients with PVR<10 Wood units (WU) and/or Qp∶Qs>1.50 while medical therapy was recommended for patients with PVR≥20 WU and/or Qp∶Qs≤1.0. For those with PVR between 10 and 20 WU and Qp∶Qs between 1.0 and 1.5, operability was determined by the cardiac surgical team after a comprehensive evaluation of clinical data, history, physical examination, chest X-ray, arterial blood gas, electrocardiogram (ECG), echocardiography and catheterization data and careful consideration of the wishes of the patient and his or her family.

### Cardiac catheterization

Cardiac catheterization was performed in all patients, using conscious sedation with continuous intravenous infusions of propofol and local analgesia, without intubation. Right atrial, pulmonary artery, and pulmonary capillary wedge pressures were measured using fluid-filled catheters. Cardiac output and pulmonary blood flow were measured by Fick's method, using assumed VO_2_ and indexed to body surface area (BSA). Assumed VO_2_ values were calculated according to the formula of LaFarge-Miettinen as follows:
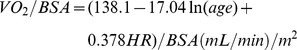
For female subjects and
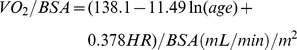
For male subjects, where age is presented in years and HR is defined as heart rate (in min). PVRI, which is PVR indexed to BSA, was calculated using the following equation:
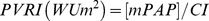
Where CI represents the cardiac index, PCWP represents pulmonary capillary wedge pressure, and WU represents Wood units. Hemodynamic measurements were made with patient breathing room air and repeated while breathing 100% oxygen, 5 to 15 L/min by facemask. PVRIO is PVRI on 100% oxygen challenge, and PVRID is the numerical difference (in Wood units) between PVRI in room air and 100% oxygen.

### Therapeutic regimen

All patients were operated under general anesthesia and moderate hypothermic cardiopulmonary bypass with cardiac arrest, and the defect was closed using standard techniques. Long-term management included the use of calcium channel blockers, phosphodiesterase 5 inhibitors (PDE5i), bosentan, cardiotonic glycosides, diuretics and warfarin, which were prescribed, where needed, at the discretion of attending physicians.

### Follow-up

Patients were followed up at 12 months postoperatively and at one year interval thereafter. They were evaluated by cardiac surgeons or pulmonologists during each follow up visit for New York Heart Association (NYHA) functional class and by six-min walk test, transthoracic echocardiography (TTE) and ECG. These variables were examined at 12 month intervals at their local pulmonary hypertension specialist centre. Long-term clinical outcome was assessed up to August 2010 by reviewing the medical files of their pulmonologists, cardiologists, and general practitioners. Baseline demographics, procedural data, and perioperative outcomes were recorded. One of the investigators reviewed the medical files of all late deaths. A separate team of research assistants prospectively collected follow-up clinical data by telephone questionnaire after the patient was discharged from the hospital.

### Statistical analysis

Basic statistical analyses were performed using the statistical software SPSS 17.0 (SPSS Inc, NUIT, and Evanston, IL, USA). Categorical data were expressed in total numbers and relative frequencies and continuous data were expressed in mean ± standard deviation (SD). The binary logistic regression models [3], [4] were chosen to predict in-hospital (early) death and total death (early death plus late death), both of which both had binary outcomes. Receiver operating characteristic (ROC) curves were used to evaluate the corresponding balance between sensitivity and specificity over a range of values for PVRI, PVRIO, PVRID, Qp∶Qs, and Rp∶Rs, and the best cutoffs of sensitivity and specificity that corresponded to the maximum area under the curve (AUC) score were determined by choosing cutoffs that engendered maximum sensitivity plus specificity. In order to assess and compare the generalization of our predictors, ten-fold cross-validation on all patients were adopted. All patients were separated into ten groups randomly at beginning. For each time, we chose one group for testing and trained a predictive model on the remaining nine-fold data, and repeated this process for ten times. Finally, we obtained ten AUC scores after ten-fold cross validation for each predictive model based on each factor. Then, Student's *t* test was used for statistical comparison of AUC scores. In addition, multivariable Cox regression was used to build predictive models for evaluating patient survival and the corresponding concordance indices were calculated by the Survcomp package in statistical language R. Hosmer-Lemeshow (H-L) test was performed for “goodness of fit” evaluation. The logistic regression model was expressed as follows:
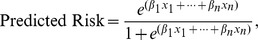
Where *x_1_, x_2_, ……* and *x_n_* denote patient preoperative risk factors (e.g., PVRI, PVRIO, and PVRID) and β_1_, β_2_… and β_n_ denote regression coefficients. The Cox regression (proportional hazards model) was expressed as follows:
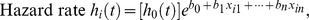
Where *x_1_, x_2_, …*, and *x_n_* denote patient preoperative risk factors (e.g., PVRI, PVRIO, and PVRID) and *b_0_*, *b_1_*, …and *b_n_* denote regression coefficients.

## Results

### Patient baseline demographic and disease characteristics

**Figure 1:**
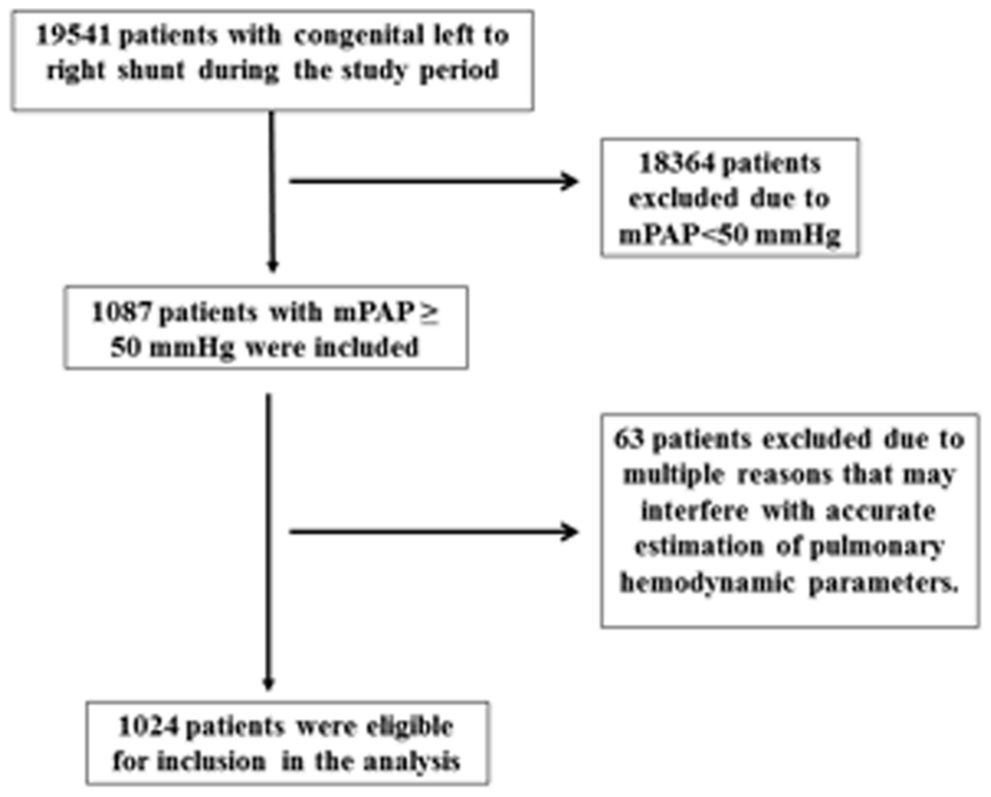
The study flow chart for 1024 patients with intracardiac shunts who had undergone surgical closure of the defects.

The study flow chart is shown in Figure 1. A total of 19451 patients with congenital intracardiac shunts received surgical treatment at our institutions throughout the study period and 1087 (5.59%) of these patients met our criteria for inclusion in this analysis. Twenty-one cases were excluded from the study because they had received heart valve repair or replacement, or other cardiac surgical procedures. Thirty-three cases were excluded because of residual heart defect after surgery, which may have impacted the severity of residual pulmonary hypertension. Nine cases were excluded because of defects such as branch pulmonary arterial stenosis, or obstruction of isolated pulmonary veins that preclude accurate calculation of PVR, Qp∶Qs, and Rp∶Rs. As a result, a total of 1024 (5.26%, including 5 patients with Down's syndrome) met the study criteria and were included in the analysis. The demographic and disease characteristics of these patients are shown in Table 1. They included 335 (34.7%) females and their mean age was 18.8±8.1 years (range, 2.4 to 44.6 years). They had a mean pulmonary arterial pressure (mPAP) of 70.2±9.2 mmHg and a PVRI of 15.5±2.6 Wood units and Qp∶Qs of 2.20±0.87. The age at the diagnosis of the cardiac defect ranged from 29 months to 45 years, and the age of operation was 30 months to 45 years. The interval between age at diagnosis and age at operation was less than three weeks.

**Table 1:** Baseline Demographic and Disease Characteristics (n = 1024).

Age, mean ± SD(interquartile range), yrs	18.8±8.1(13.2–23.9)
Age older than 18 yrs, n (%)	543(53)
Female, n (%)	335(34.7)
Primary CHD	
ASD, n (%)	126 (12.3)
VSD, n (%)	576 (56.2)
PDA, n (%)	123(12.1)
ASD+VSD, n (%)	74 (7.2)
ASD+PDA, n (%)	23 (2.3)
VSD+PDA, n (%)	71 (7.0)
ASD+VSD+PDA, n (%)	20 (2.0)
APW, n (%)	11 (1.1)
Shunt level	
NYHA functional class ≥III, n (%)	237(23.1)
6MWD (m)	386±116.6
Hemoglobin (g/L)	179±21
LVEF (%)	61.6±7.9
SaO_2_ (%)	91.3±3.4
Right atrial pressure (mmHg)	10.7±2.7
mPAP (mm Hg)	70.2±9.2
SPAP/SBP	0.99±0.078
PVRI (Wood Units)	15.5±2.6
Qp∶Qs, mean ± SD (interquartile range)	2.20±0.87(1.56–2.65)
Rp∶Rs, mean + SD (interquartile range)	0.71±0.17(0.59–0.83)

6MWD, 6 min walk distance; APW, aorto-pulmonary windows; ASD, atrial septal defect; CHD, congenital heart disease; LVEF, left ventricular ejection fraction; mPAP, mean pulmonary artery pressure; PDA, patent ductus arteriosus; PVRI, pulmonary vascular resistance, indexed to body surface area; Qp∶Qs, pulmonary to systemic flow ratio; Rp∶Rs, ratio of pulmonary and systemic vascular resistance; SPAP, systolic pulmonary artery pressure; SPAP/SBP, pulmonary to systemic systolic artery pressure ratio; TTE, transthoracic echocardiography; VSD, ventricular septal defect.

### Early and late mortality

**Figure 2:**
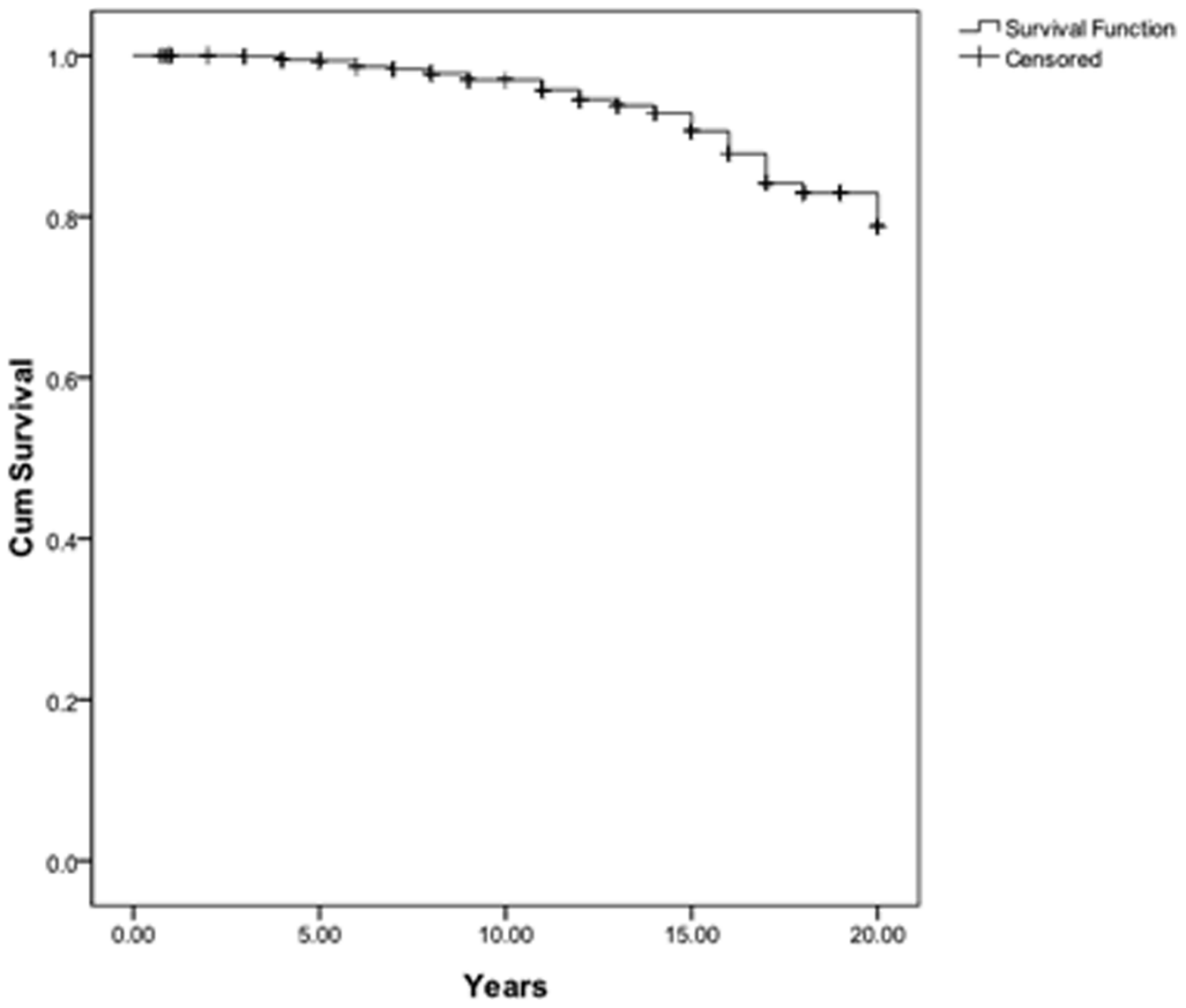
The Kaplan-Meier survival curve after the shunt closure procedure for patients with congenital systemic-to-pulmonary shunts and increased pulmonary vascular resistance.

Three hundred and twenty nine (32.1%) patients experienced pulmonary hypertensive crisis and acute heart failure, and 61 succumbed to the crisis despite therapy with PGE_1_, iloprost and inhaled NO, and mechanical ventilatory support. Thus, the in-hospital surgical mortality was 5.96% (61/1024) and 963 patients, including 5 patients with Down syndrome, were available for follow up. The mean duration of the follow-up period was 8.5±5.5 years (range, 0.7 to 20 years). One hundred sixty-nine patients (17.5%) received medication with calcium channel blockers, 23 (2.4%) with PDE5i, 6 (0.63%) with bosentan, 138 (14.3%) with glycosides, 139 (14.4%) with diuretics, and 15 (1.6%) with warfarin. Forty-six late deaths were reported from chronic right heart failure (23), arrhythmia (8), acute pulmonary hypertension (7), lung infection (4), hemoptysis (3), and brain abscess (1). The late postoperative mortality rate was 4.49%. The Kaplan-Meier survival curve for late death after shunt closure is depicted in Figure 2. The cumulative survival stood at 95.7% at 10 years and 81.7% at 20 years.

### Pulmonary vascular hemodynamic parameters predict risks for early and late mortality

We further examined whether survival was associated with any of the demographic or pulmonary hemodynamic characteristics of these patients. We found a statistically significant difference in PVRI, PVRIO, PVRID, Qp∶Qs, and Rp∶Rs in early survivors and early non-survivors (*P*<0.001) (Table 2). ROC curve analysis further revealed that the use of PVRI<18.1 WU.m^2^, PVRIO<11.1 WU.m^2^, PVRID>7.3 WU.m^2^ on 100% oxygen, Qp∶Qs<1.34, and Rp∶Rs>0.82 as pulmonary hemodynamic criteria for surgical closure of intracardiac shunts provided the optimal positive and negative predictive value, sensitivity, specificity, and accuracy for in-hospital death (see Fig. S1 and Table S1 in File S1). On the other hand, PVRI>17.6 WU.m^2^, PVRIO>10.3 WU.m^2^, PVRID<7.3 WU.m^2^ on 100% oxygen, Qp∶Qs<1.55, and Rp∶Rs>0.83 provided the optimal positive and negative predictive value, sensitivity, specificity, and accuracy for total death, respectively (see Fig. S2 and Table S2 in File S1). In addition, PVRIO better predicted in-hospital and total death than PVRI, PVRID, Qp∶Qs, and Rp∶Rs (*P*<0.000 in all).

**Table 2:** Pulmonary Vascular Hemodynamic Risk Factors in Early Survivors and Non-survivors of Surgical Patients with Intracardiac Shunts.

	Early Death (n = 61)	Early Survival (n = 963)	P
Age at operation, mean ± SD, yrs, (interquartile range)	19.8±9.1 (11.1–27.7)	18.8±8.1 (13.3–23.8)	0.238
Age at operation, median, yrs	20.9	18.5	
PVRI (WU)	20.25±2.20	15.17±2.25	0.000
PVRIO (WU)	16.63±3.20	7.15±2.58	0.000
PVRID (WU)	3.63±1.83	8.01±1.01	0.000
Rp∶Rs	1.02±0.14	0.69±0.15	0.000
Qp∶Qs	1.19±0.22	2.26±0.86	0.000

PVRI, pulmonary vascular resistance index; PVRIO, pulmonary vascular resistance index on pure oxygen challenge; PVRID, difference between PVRI and PVRIO; Qp∶Qs, pulmonary to systemic flow ratio; Rp∶Rs, ratio of pulmonary and systemic vascular resistance; WU, Wood units.

The above findings suggested that these individual parameters predicted in-hospital death and total death risk. We were further interested in investigating whether these parameters in combination could predict early and late mortality. We firstly built logistic regression for each factor, and then used binary logistic regression with likelihood ratio-based forward stepwise strategy to predict early death and late death by automatically selecting useful factors from all the factors. The results for each single factor and for the final model for in-hospital death and late death prediction are shown in the supplementary tables (Table S3 and Table S4 in File S1, respectively) with corresponding H-L test for “goodness of fit” evaluation. The results showed that PVRIO and PVRID were the most important risk factors for both in-hospital death and late death and Qp∶Qs was another important factor for predicting in-hospital death.

Using late death data, we asked how these hemodynamic factors affected the prediction of survival time. Using ROC curves to obtain the optimal thresholds as separate criteria for predicting late death as we did in predicting early death and total death (Figure S1 and S2), we found that PVRIO also better predicted late death than PVRI, PVRID, Qp∶Qs, and Rp∶Rs (*P*<0.000 in all) (also see Table S5 in File S1). Given the abundance of available survival data, we then used more advanced methods with consideration of censoring information to predict late death. We built Cox regression models based on each individual factor to predict late death and used concordance index [5] to assess their performance. The results show that, of the four parameters, PVRIO displayed the best performance (Table S6 in File S1). We further selected all the variables together to build a forward stepwise Cox regression model (based on likelihood ratio) to predict late death. We found that the final model only included PVRID and Qp∶Qs, as shown in the formula: 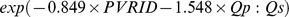, and the corresponding concordance index was equal to 0.796±0.041 with *P*<0.000.

We further analyzed the performance of hemodynamic parameters in predicting survival of patients with intracardiac shunts (see Table S7 in File S1 for detailed characteristics). We built three Cox regression models (with a likelihood ratio-based forward stepwise strategy) separately for patients with pre-tricuspid shunt, post-tricuspid shunt or both. The final model for the ASD subgroup contained only PVRID (with coefficient of −1.284); and the same was true for the VSD subgroup (coefficient of −1.015), while for the ASD and VSD subgroup, the final model contained two hemodynamic parameters PVRID (with coefficient of −0.851) and Qp∶Qs (with coefficient of −1.519). The PVRID was thus the most important predictor for survival events no matter which subgroups the patient belongs to, and gender was not a significant factor for any of the subgroups.

## Discussion

Pulmonary arterial hypertension (PAH) commonly arises in patients with CHD and its management may involve surgical correction of the cardiac defect and/or treatment of PAH, depending on the underlying cardiac defect and status of disease progression. The timing of intervention in patients with PAH-CHD is important, but the optimum time is sometimes difficult to determine, with limited robust data to support an informed management decision [6]. In patients with congenital intracardiac shunting lesions, once advanced pulmonary vascular occlusive disease is established, closure of the defect may markedly shorten survival relative to leaving the defect open as in Eisenmenger syndrome [7]. This often remains a critical issue for older patients and for children 2 or 3 years of age who have a complete atrioventricular canal or Down syndrome. It is therefore imperative to assess the risk of post-shunt closure pulmonary hypertension in all patients before referring them for operation. Many papers have been published relating various hemodynamic variables to outcome after shunt closure, but considerable uncertainty remains regarding which baseline variables and what values associated with these variables confer a favorable risk-benefit ratio for operation [1], [2]. The utility of pulmonary vasodilators in revealing the likelihood of high postoperative PVR is also uncertain. Two studies suggest that PVRI≤7 WU with a vasodilator (tolazoline) conferred suitability for operation [8], [9], but a third study found that PVRI (with iNO and 100% O_2_) of <5.3 was more suitable [1]. Still others indicate that Rp∶Rs with vasodilators is a better indicator of operative risk [1]. One study found that vasodilation with 100% oxygen did not guarantee low PVR after defect closure [10].

There are multiple reasons for this uncertainty, including considerable biological variability in the response of the pulmonary circulation to intracardiac shunting. Previous papers report different hemodynamic variables (e.g., Rp∶Rs vs. PVR, and PVR vs. indexed PVR), in patients of different ages, with different durations of follow-up. How outcomes are reported varies, and it is sometimes unclear whether postoperative death was related to PAH or some other factors. Some papers reflect relatively early experience in the era of open heart surgery before effective management of postoperative PAH was available [11], [12]. Finally, available reports include only a relatively small number of patients (<100 patients/report) with septal defects and significantly increased PAP and PVR [1], [8]–[18]. Mean duration of follow-up is often not clearly given, but is usually much less than 10 years [8], [11], [16], [18]. Except the study by Balzer *et al.*, these reports lack a sufficient number of patients and detailed follow-up to permit a formal and thorough statistical analysis of the predictive power of multiple hemodynamic variables to predict outcome after operation.

In this study, we report our retrospective review of clinical and hemodynamic data of a large cohort of patients with intracardiac shunts who had undergone surgical closure of cardiac defects. The longest follow up of these patients is 20 years with a mean duration follow up of 8.5±5.5 years. The abundance of available data from the data set and the length of follow up allowed us to carry out a considerably more extensive analysis of hemodynamic predictors of survival for these patients. Notably, we found that the risk of early or late death was unrelated to patient age. It has been clearly documented that very young patients (<∼2 years old) are much less likely to have persistently increased PVR after VSD repair than older children [13], [19]. It appears that beyond the first few years of life, age is not a significant discriminator of outcome (at least in the first ∼20 years of life). Our findings support the idea that some adolescents with high pressure shunts can safely undergo repair. Though this is not a new finding, our finding carries more weight given the much larger number of patients in our study than any previous reports [17], [18], [20].

We further show that PVRIO<10.3 WU.m^2^ and PVRID>7.3 WU.m^2^ best predicted survival. Perhaps not surprisingly, and consistent with previous reports [1], [14], Rp∶Rs is different in survivors and non-survivors, with Rp∶Rs of <0.83 being associated with a good outcome, although it is not as good a predictor of outcome as PVRIO or PVRID. Qp∶Qs is also not as powerful a predictor, although it is notable that the average Qp∶Qs in survivors is substantially different from non-survivors. The final model for predicting survival from late death after the shunt closure procedure only includes PVRID and Qp∶Qs, suggesting that the combination of informative factors can yield much better performance and the final model could predict the survival time quite well. Since measurement of PVR is fraught with potential error, taking into account all 4 variables, not only PVRIO and PVRID, but also PVRID and Qp∶Qs, is important in determining whether to proceed an operation.

The iNO, with or without enhanced inspired oxygen, is usually used for vasodilator testing today [1], [21]. When we first commenced this protocol, iNO was not available for such use, for the sake of consistency; we report here only our experience with 100% oxygen. Despite the use of a seemingly sub-optimal vasodilator regimen, we found that PVRID with 100% fiO_2_ is a sensitive and specific predictor of postoperative mortality. Our finding that a fall in PVRI with a vasodilator of <17.6 Wood units suggesting a low likelihood of postoperative death is consistent with other studies [1], [8], [9], but not with that by Lock, *et al* [10]. In addition, we found little difference between patients with ASD and those with VSD regarding survival predictors. PVRID is thus the most important predictor for survival events no matter which subgroups (i.e., post-tricuspid shunt, pre-tricuspid shunt and both) the patient belongs to, and gender is not a significant factor for any of the subgroups.

This report has several limitations. As noted, our vasodilator testing protocol no longer reflects the most current practice; it is possible that the addition of iNO may further enhance predictive power of the change in PVR with vasodilation. In addition, we used assumed oxygen consumption, doubtlessly introducing some errors into calculations of pulmonary blood flow and derived variables. Furthermore, we only reported the outcomes of patients who had defect closure; it is likely that some patients who did not undergo operation would have done well with repair. Moreover, our study is only weakly powered to identify patients who develop findings related to progressive PAH very late (>10 years postoperatively).

In summary, we have taken advantage of a very large clinical data set to ask which pulmonary vascular hemodynamic parameters best predict survival in patients with congenital shunting lesions and high preoperative PAP after surgical repair. We found that preoperative PVRI, PVRIO, Qp∶Qs, Rp∶Rs, and change in PVRI with 100% O_2_ can all predict in-hospital death and total death, with PVRIO being the most sensitive and specific predictor. A very similar result was obtained for predicting late death (by evaluating the survival time). Despite the very high degree of sensitivity and specificity of PVRID in predicting postoperative survival, it is important that this only occurs when multiple factors have been taken into account in judging suitability for repair of patients with intracardiac shunting lesions.

## Conclusion

In conclusion, our study demonstrates that all 4 pulmonary hemodynamic variables, PVRI, PVRIO, PVRID and Qp∶Qs, should be considered in deciding surgical closure of congenital septal defects and a PVRIO<10.3 WU.m^2^ and PVRID>7.3 WU.m^2^ on 100% oxygen are associated with a favorable risk benefit profile for the procedure and suggests that closure of congenital septal defects can be undertaken with acceptable risk.

## Supporting Information

Figure S1 **ROC curves for PVRI, PVRIO, PVRID, Rp∶Rs, and Qp∶Qs as predictors of early death.** We chose cutoff points for operability for the 5 variables by inspecting the ROC curves to identify the point where specificity plus sensitivity was found to be maximal. PVRI, pulmonary vascular resistance index; PVRID, difference between PVRI and PVRIO; PVRIO, pulmonary vascular resistance index on pure oxygen challenge; Qp∶Qs, pulmonary to systemic flow ratio; Rp∶Rs, ratio of pulmonary and systemic vascular resistance; WU, Wood units.
(TIF)

Figure S2 **ROC curves for PVRI, PVRIO, PVRID, Rp∶Rs, and Qp∶Qs as predictors of total death (early and late death).** We picked cutoff points for operability for the 5 variables by inspecting the ROC curves to identify the point where specificity plus sensitivity was found to be maximal. PVRI, pulmonary vascular resistance index; PVRID, difference between PVRI and PVRIO; PVRIO, pulmonary vascular resistance index on pure oxygen challenge; Qp∶Qs, pulmonary to systemic flow ratio; Rp∶Rs, ratio of pulmonary and systemic vascular resistance; WU, Wood units.
(TIF)

File S1 Table S1 in File S1. ROC Characteristics of Pulmonary Vascular Hemodynamic Parameters as Predictors of Early Mortality in Surgical Patients with Intracardiac Shunts. Table S2 in File S1. ROC characteristics of Pulmonary Vascular Hemodynamic Parameters as Predictors of Total (Early and Late) Mortality in Surgical Patients with Intracardiac Shunts. Table S3 in File S1. Odds Ratios with 95% Confidence Intervals (CI) for Single Factor-based Risk Models. Table S4 in File S1. Multivariate Logistic Regression Model Performance. Table S5 in File S1. ROC characteristics of Pulmonary Vascular Hemodynamic Parameters as Predictors of Late Mortality in Surgical Patients with Intracardiac Shunts. Table S6 in File S1. The Performance of Cox Regression Model for Predicting Late Deaths Built on Different Pulmonary Vascular Hemodynamic Parameters. Table S7 in File S1. Characteristics of Patients with Pre-tricuspid and Post-tricuspid shunts
(DOC)
