# Supplementary figures and images for: Patients with Congenital Systemic-to-Pulmonary Shunts and Increased Pulmonary Vascular Resistance: What Predicts Postoperative Survival?
Source: PLoS One. 2014 Jan 8;9(1):e83976. doi: 10.1371/journal.pone.0083976 (PMC3885539; doi:10.1371/journal.pone.0083976)

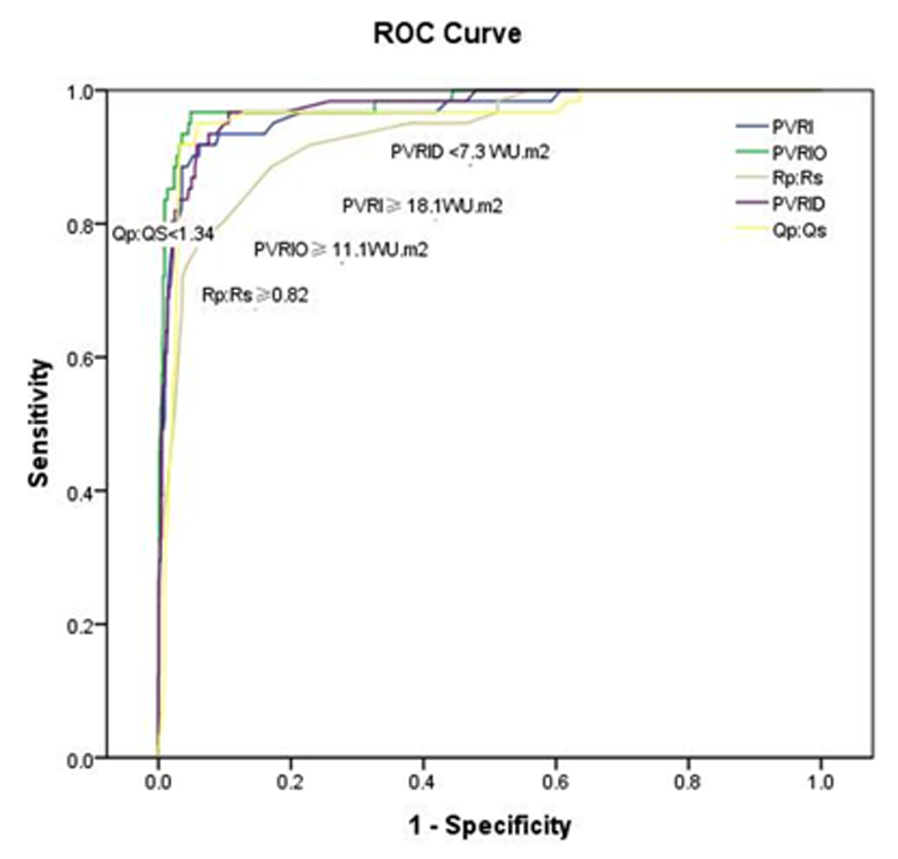

Supplement: Figure S1 — ROC curves for PVRI, PVRIO, PVRID, Rp∶Rs, and Qp∶Qs as predictors of early death. We chose cutoff points for operability for the 5 variables by inspecting the ROC curves to identify the point where specificity plus sensitivity was found to be maximal. PVRI, pulmonary vascular resistance index; PVRID, difference between PVRI and PVRIO; PVRIO, pulmonary vascular resistance index on pure oxygen challenge; Qp∶Qs, pulmonary to systemic flow ratio; Rp∶Rs, ratio of pulmonary and systemic vascular resistance; WU, Wood units. (TIF) [file pone.0083976.s001.tif]

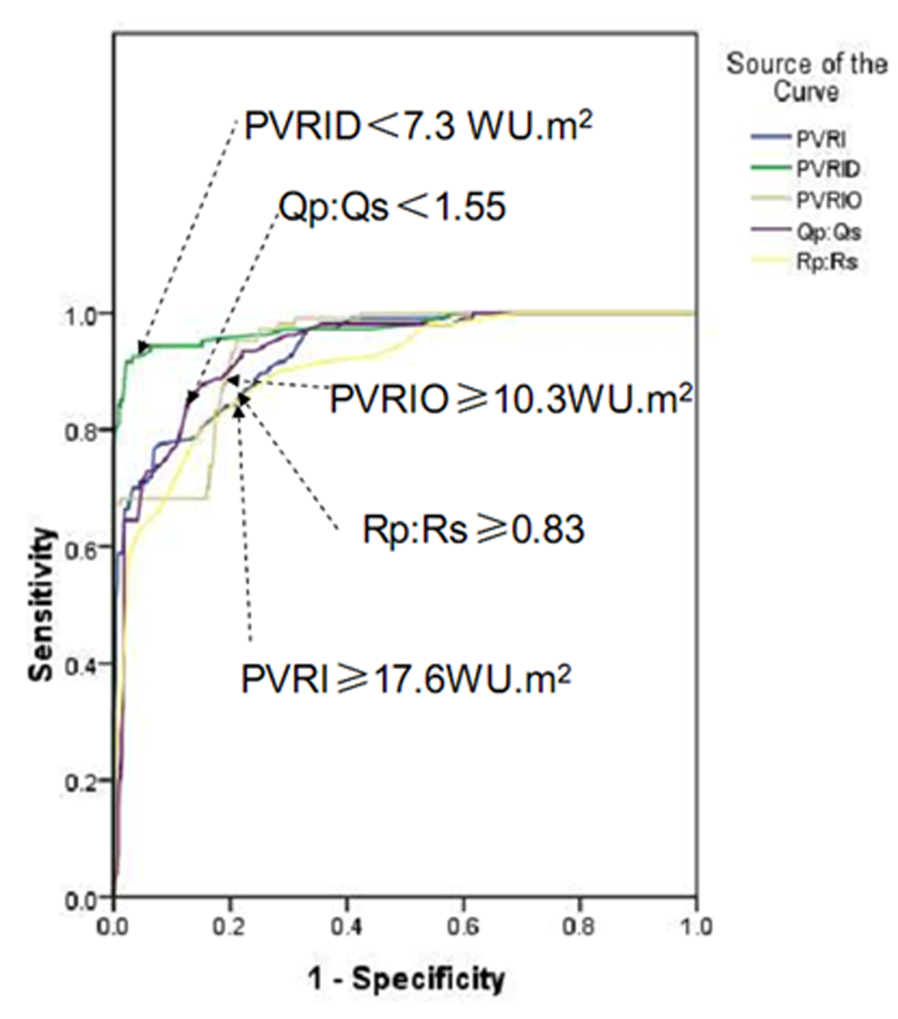

Supplement: Figure S2 — ROC curves for PVRI, PVRIO, PVRID, Rp∶Rs, and Qp∶Qs as predictors of total death (early and late death). We picked cutoff points for operability for the 5 variables by inspecting the ROC curves to identify the point where specificity plus sensitivity was found to be maximal. PVRI, pulmonary vascular resistance index; PVRID, difference between PVRI and PVRIO; PVRIO, pulmonary vascular resistance index on pure oxygen challenge; Qp∶Qs, pulmonary to systemic flow ratio; Rp∶Rs, ratio of pulmonary and systemic vascular resistance; WU, Wood units. (TIF) [file pone.0083976.s002.tif]
