# Supplementary material for: Patients with Congenital Systemic-to-Pulmonary Shunts and Increased Pulmonary Vascular Resistance: What Predicts Postoperative Survival?
Source: PLoS One. 2014 Jan 8;9(1):e83976. doi: 10.1371/journal.pone.0083976 (PMC3885539; doi:10.1371/journal.pone.0083976)
Supplement: File S1 — Table S1 in File S1. ROC Characteristics of Pulmonary Vascular Hemodynamic Parameters as Predictors of Early Mortality in Surgical Patients with Intracardiac Shunts. Table S2 in File S1. ROC characteristics of Pulmonary Vascular Hemodynamic Parameters as Predictors of Total (Early and Late) Mortality in Surgical Patients with Intracardiac Shunts. Table S3 in File S1. Odds Ratios with 95% Confidence Intervals (CI) for Single Factor-based Risk Models. Table S4 in File S1. Multivariate Logistic Regression Model Performance. Table S5 in File S1. ROC characteristics of Pulmonary Vascular Hemodynamic Parameters as Predictors of Late Mortality in Surgical Patients with Intracardiac Shunts. Table S6 in File S1. The Performance of Cox Regression Model for Predicting Late Deaths Built on Different Pulmonary Vascular Hemodynamic Parameters. Table S7 in File S1. Characteristics of Patients with Pre-tricuspid and Post-tricuspid shunts (DOC) [file pone.0083976.s003.doc]

# Patients with Congenital Systemic-to-Pulmonary Shunts and Increased Pulmonary Vascular Resistance: What predicts postoperative survival?

# Hui-Li Gan, MD, PhD,*, Jian-Qun Zhang, MD, Qi-Wen Zhou, MD, Lei Feng, MD, Fei Chen, MD, and Yi Yang, MD

# Department of Cardiac Surgery, Beijing Anzhen Hospital, Capital Medical University, Beijing Institute of Heart, Lung and Blood Vessel Diseases, Beijing 100029, China

# *Corresponding Author: Hui-Li Gan, MD, PhD, Department of Cardiac Surgery, Beijing Anzhen Hospital, Capital Medical University, Beijing Institute of Heart, Lung and Blood Vessel Diseases, Beijing 100029, China (Tel: +86-10-64456885; Fax: +86-10-62244207 Email:ganhuili@hotmail.com)

Supplementary Materials

Table S1-7

Figure S1,S2

| **Table S1. ROC Characteristics of** **Pulmonary Vascular Hemodynamic Parameters as Predictors of Early Mortality in Surgical Patients with Intracardiac Shunts** | | | | | | | | | | |
| --- | --- | --- | --- | --- | --- | --- | --- | --- | --- | --- |
|  | PVRI 18.1 WU | | PVRIO 11.1WU | | PVRID 7.3 WU | | Qp:Qs 1.34 | | Rp:Rs 0.82 | |
|  | Death | Survival | Death | Survival | Death | Survival | Death | Survival | Death | Survival |
| ≥ the hemodynamic variable | 56 | 66 | 59 | 47 | 4 | 890 | 58 | 58 | 7 | 799 |
| < the hemodynamic variable | 5 | 897 | 2 | 916 | 57 | 73 | 3 | 905 | 54 | 164 |
| Sensitivity, % | 91.3 | | 96.7 | | 93.4 | | 95.1 | | 88.5 | |
| Specificity, % | 93.1 | | 95.179.1 | | 92.4 | | 94 | | 83 | |
| Accuracy, % | 93.1 | | 95.2 | | 92.5 | | 94 | | 83.3 | |
| Positive Predictive Value, % | 45.9 | | 55.7 | | 43.8 | | 50 | | 24.8 | |
| Negative Predictive Value, % | 99.4 | | 99.8 | | 99.6 | | 99.7 | | 99.1 | |
| Area Under the Curve (AUC) | 0.965±0.0.016  (0.933,0.998) | | 0.980±0.013  (0.956,1.0) | | 0.972±0.015  (0.944,1) | | 0.960±0.018  (0.925, 0.994) | | 0.933±0.022  (0.889, 0.977) | |
| *P* value | 0.000 | | 0.000 | | 0.000 | | 0.000 | | 0.000 | |
| The difference between the five curves PVRIO better predicts in-hospital than other 4 variables (T-test for PVRIO vs. PVRI, PVRID, Qp:Qs,and Rp:Rs, the p-values are all < 0.000 respectively). (95% CI). CI, confidence interval; PVRI, pulmonary vascular resistance index; PVRIO, pulmonary vascular resistance index on pure oxygen challenge; PVRID, difference between PVRI and PVRIO; Qp:Qs, pulmonary to systemic flow ratio; Rp:Rs, ratio of pulmonary and systemic vascular resistance; WU, Wood units. | | | | | | | | | | |

| **Table S2. ROC characteristics of Pulmonary Vascular Hemodynamic Parameters as Predictors of Total (Early and Late) Mortality in Surgical Patients with Intracardiac Shunts** | | | | | | | | | | |
| --- | --- | --- | --- | --- | --- | --- | --- | --- | --- | --- |
|  | PVRI 17.6 WU | | PVRIO 10.3WU | | PVRID 7.3 WU | | Rp:Rs 0.83 | | Qp:Qs 1.55 | |
|  | Death | Survival | Death | Survival | Death | Survival | Death | Survival | Death | Survival |
| ≥the hemodynamic variable | 86 | 139 | 99 | 38 | 8 | 886 | 84 | 134 | 13 | 774 |
| < the hemodynamic variable | 21 | 779 | 8 | 879 | 99 | 31 | 23 | 783 | 94 | 143 |
| Sensitivity, % | 80.4 | | 92.5 | | 92.5 | | 78.5 | | 87.9 | |
| Specificity, % | 84.8 | | 95.9 | | 96.6 | | 85.4 | | 84.4 | |
| Accuracy, % | 84.4 | | 95.5 | | 96.2 | | 84.7 | | 84.8 | |
| Positive Predictive Value, % | 38.2 | | 72.3 | | 76.2 | | 38.5 | | 39.7 | |
| Negative Predictive Value, % | 97.4 | | 99.1 | | 99.1 | | 97.1 | | 98.3 | |
| Area Under the Curve (AUC) | 0.931±0.017  (0.898, 0.965) | | 0.985±0.0082  (0.969,1) | | 0.974±0.01  (0.953,0.995) | | 0.90±0.02  (0.86, 0.939) | | 0.933±0.017  (0.90, 0.966) | |
| *P* value | 0.000 | | 0.000 | | 0.000 | | 0.000 | | 0.000 | |
| (95% CI). CI, confidence interval; PVRI, pulmonary vascular resistance index; PVRIO, pulmonary vascular resistance index on pure oxygen challenge; PVRID, difference between PVRI and PVRIO; Qp:Qs, pulmonary to systemic flow ratio; Rp:Rs, ratio of pulmonary and systemic vascular resistance; WU, Wood units. | | | | | | | | | | |

**Table S3. Odds Ratios with 95% Confidence Intervals (CI) for Single Factor-based Risk M**odels

| *Variables* | *Total Death (95% CI)* | *Early Death (95% CI)* | *Late Death (95% CI)* |
| --- | --- | --- | --- |
| PVRI | 0.885 (0.875,0.896) | 0.852(0.840,0.865) | 0.830(0.815,0.846) |
| PVRIO | 0.853(0.837,0.870) | 0.789(0.770,0.809) | 0.724(0.701,0.749) |
| PVRID | 0.697(0.673,0.722) | 0.630(0.599,0.662) | 0.644(0.614,0.675) |
| Qp:Qs | 0.279(0.245,0.317) | 0.189(0.157,0.226) | 0.192(0.159,0.231) |
| PVR | 0.857(0.843,0.870) | 0.814(0.798,0.831) | 0.780(0.761,0.800) |
| Rp:Rs | 0.083(0.064,0.107) | 0.036(0.026,0.049) | 0.019(0.013,0.028) |

PVRI, pulmonary vascular resistance index; PVRIO, pulmonary vascular resistance index on pure oxygen challenge; PVRID, difference between PVRI and PVRIO; Qp:Qs, pulmonary to systemic flow ratio; Rp:Rs, ratio of pulmonary and systemic vascular resistance.

**Table S4. Multivariate Logistic Regression Model Performance**

|  | AUC score | H-L test | H-L Test *P* value | Factors with Odds Ratios (95% CI) |
| --- | --- | --- | --- | --- |
| Early death | 0.977 | 101.89 | <0.0001 | PVRIO: 1.152(1.37,1.759)  PVRID: 0.753(0.591,0.960)  Qp:Qs: 0.014(0.002,0.104) |
| Late death | 0.985 | 4.071 | 0.851 | PVRIO:2.509(1.889,3.333)  PVRID:0.192 (0.126.0.293) |
| Total death | 0.989 | 5.328 | 0.722 | PVRID: 2.554(1.99,3.278)  PVRIO: 0.2(0.139,0.288) |

AUC: area under the curve; H-L, Hosmer-Lemeshow; PVRI, pulmonary vascular resistance index; PVRIO, pulmonary vascular resistance index on pure oxygen challenge; PVRID, difference between PVRI and PVRIO; Qp:Qs, pulmonary to systemic flow ratio; Rp:Rs, ratio of pulmonary and systemic vascular resistance.

| **Table S5.** **ROC characteristics of Pulmonary Vascular Hemodynamic Parameters as Predictors of Late Mortality in Surgical Patients with Intracardiac Shunts** | | | | | | | | | | |
| --- | --- | --- | --- | --- | --- | --- | --- | --- | --- | --- |
|  | PVRI 16.6 WU | | PVRIO 9.9 WU | | PVRID 6.9 WU | | Rp:Rs 0.77 | | Qp:Qs 1.67 | |
|  | Death | Survival | Death | Survival | Death | Survival | Death | Survival | Death | Survival |
| ≥ the hemodynamic variable | 38 | 246 | 43 | 78 | 4 | 894 | 38 | 255 | 5 | 711 |
| < the hemodynamic variable | 8 | 671 | 3 | 839 | 42 | 23 | 8 | 622 | 41 | 206 |
| Sensitivity, % | 82.6 | | 93.5 | | 91.3 | | 82.6 | | 89.1 | |
| Specificity, % | 73.2 | | 91.5 | | 97.5 | | 72.2 | | 77.5 | |
| Accuracy, % | 73.6 | | 91.6 | | 97.2 | | 86.4 | | 78.1 | |
| Positive Predictive Value, % | 13.4 | | 35.5 | | 64.6 | | 13.4 | | 16.6 | |
| Negative Predictive Value, % | 98.8 | | 99.6 | | 99.6 | | 98.8 | | 99.3 | |
| Area Under the Curve | 0.876±0.033  (0.811, 0.942) | | 0.982±0.014  (0.955,1) | | 0.96±0.02  (0.919,0.999) | | 0.844±0.037  (0.772, 0.915) | | 0.891±0.032  (0.829, 0.953) | |
| *P* value | 0.000 | | 0.000 | | 0.000 | | 0.000 | | 0.000 | |
| (95% CI). CI, confidence interval; AUC: area under the curve; PVRI, pulmonary vascular resistance index; PVRIO, pulmonary vascular resistance index on pure oxygen challenge; PVRID, difference between PVRI and PVRIO; Qp:Qs, pulmonary to systemic flow ratio; Rp:Rs, ratio of pulmonary and systemic vascular resistance; WU, Wood units. | | | | | | | | | | |

**Table S6. The Performance of Cox Regression Model for Predicting Late Deaths Built on Different Pulmonary Vascular Hemodynamic Parameters**

| *Parameters* | *C-index* | *SD* | *P value* |
| --- | --- | --- | --- |
| PVRI | 0.578 | 0.031 | 0.0061 |
| PVRIO | 0.769 | 0.033 | <0.000 |
| PVRID | 0.718 | 0.059 | 0.00010 |
| Rp:Rs | 0.465 | 0.040 | 0.190 |
| Qp:Qs | 0.607 | 0.027 | <0.000 |

C-index, concordance index; PVRI, pulmonary vascular resistance index; PVRIO, pulmonary vascular resistance index on pure oxygen challenge; PVRID, difference between PVRI and PVRIO; Qp:Qs, pulmonary to systemic flow ratio; Rp:Rs, ratio of pulmonary and systemic vascular resistance.

**Table S7. Characteristics of Patients with Pre-tricuspid and Post-tricuspid shunts**

|  | Pre-tricuspid | Post-tricuspid | Both |
| --- | --- | --- | --- |
| No. of patients, n (%) | 126 (12.3) | 781(76.3) | 117 (11.4) |
| Age, mean + SD | 25.6± 9.3 | 18±7.6 | 17±7 |
| (interquartile range), yrs | (20.1-31) | (12.8-22.8) | (12.2-21.8) |
| Female, n (%) | 65 (51.6) | 248 (31.8) | 42 (35.9) |
| Body weight | 51±12 | 44±13 | 43±12 |
| Body surface area | 1.491±0.237 | 1.343±0.272 | 1.326±0.267 |
| 6MWD (m) | 376±117 | 385±117 | 406±111 |
| Hemoglobin (g/L) | 17.9±2 | 18±2.1 | 17.6±2.2 |
| LVEF (%) | 61±8 | 62±8 | 62±8 |
| PaO_2_ | 73±9 | 73±9 | 73±9 |
| SaO_2_ | 91±4 | 91±3 | 91±3 |
| Right atrial pressure (mmHg) | 11±3 | 11±3 | 11±2 |
| mPAP | 69±9.4 | 70.6±9.4 | 68.9±7.9 |
| PVRI (Wood Units) | 15.2±2.4 | 15.5±2.6 | 15.4±2.7 |
| Qp:Qs, mean + SD | 2.25±0.77 | 2.18±0.88 | 2.29±0.9 |
| Rp: Rs, mean + SD | 0.689±0.17 | 0.717±0.169 | 0.691±0.179 |

Note: Pre-tricuspid:ASD; Post-tricuspid: VSD, PDA, APW, VSD+PDA; Both: ASD+VSD, ASD+PDA,ASD+VSD+PDA; 6MWD, 6 min walk distance; APW, aorto-pulmonary windows; ASD, atrial septal defect; LVEF, left ventricular ejection fraction; mPAP, mean pulmonary artery pressure; PDA, patent ductus arteriosus; PVRI, pulmonary vascular resistance index; Qp:Qs, pulmonary to systemic flow ratio; Rp:Rs, ratio of pulmonary and systemic vascular resistance; VSD, ventricular septal defect.

**Figure Legends**

**Figure S1.** ROC curves for PVRI, PVRIO, PVRID, Rp:Rs, and Qp:Qs as predictors of early death. We chose cutoff points for operability for the 5 variables by inspecting the ROC curves to identify the point where specificity plus sensitivity was found to be maximal. PVRI, pulmonary vascular resistance index; PVRID, difference between PVRI and PVRIO; PVRIO, pulmonary vascular resistance index on pure oxygen challenge; Qp:Qs, pulmonary to systemic flow ratio; Rp:Rs, ratio of pulmonary and systemic vascular resistance; WU, Wood units.

**Figure S2.** ROC curves for PVRI, PVRIO, PVRID, Rp:Rs, and Qp:Qs as predictors of total death (early and late death). We picked cutoff points for operability for the 5 variables by inspecting the ROC curves to identify the point where specificity plus sensitivity was found to be maximal. PVRI, pulmonary vascular resistance index; PVRID, difference between PVRI and PVRIO; PVRIO, pulmonary vascular resistance index on pure oxygen challenge; Qp:Qs, pulmonary to systemic flow ratio; Rp:Rs, ratio of pulmonary and systemic vascular resistance; WU, Wood units.
